# Enhancing Strength and Sustainability: Evaluating Glass and Basalt Fiber-Reinforced Biopolyamide as Alternatives for Petroleum-Based Polyamide Composite

**DOI:** 10.3390/polym15163400

**Published:** 2023-08-14

**Authors:** Dariusz Bednarowski, Patrycja Bazan, Stanisław Kuciel

**Affiliations:** 1 Chair of Material Engineering and Physics, Cracow University of Technology, 31-155 Kraków, Poland; dariusz.bednarowski@gmail.com (D.B.); stanislaw.kuciel@pk.edu.pl (S.K.); 2 ABB Corporate Technology Center, 31-038 Kraków, Poland

**Keywords:** biopolyamide, basalt fibers, glass fibers, composites

## Abstract

This study aims to analyze strength properties and low-cycle dynamic tests of composite materials modified with glass and basalt fibers. Biopolyamide 4.10 was used as the matrix, and the fiber contents were 15, 30, and 50% by weight. Static tensile tests, impact tests, and determination of mechanical hysteresis loops were carried out as strength tests. The length of the fibers in the produced composites and their processing properties were determined. The composite materials were compared with commercially available glass fiber-reinforced composites with 30 and 50% fiber contents. The results showed that such composites can successfully replace composite materials based on petroleum-based polymeric materials, providing high strength properties and reducing the negative environmental impact by using renewable sources. Composites with 30% basalt fiber composition were characterized by higher tensile strength by about 60% compared to commercially available composites with 30% glass fiber composition and an almost doubly increased Young’s modulus. Increasing the content of basalt fibers to 50% results in a further increase in strength properties. Despite the lower tensile strength compared to polyamide 6 with 50% glass fiber content, basalt fibers provided an approximately 10% higher modulus of elasticity.

## 1. Introduction

Polymer composites are included in the engineering materials group. Introducing fillers in the form of fibers and particles into the polymer matrix can improve processing, as well as thermal, strength, and performance properties. Fiberglass and carbon fibers are widely used as reinforcements in the thermoplastic matrix because they provide an excellent balance of mechanical properties and economic characteristics [1,2].

Glass fiber-reinforced polymer composites can be produced by hand lay-up, spray lay-up, pultrusion lay-up, pre-impregnated lay-up, compression molding, injection molding, or infusion molding. Glass fibers have excellent properties such as high strength, flexibility, stiffness, and chemical resistance. They are produced as rovings, chopped strands, yarns, fabrics, and mats. Each type of glass fiber has unique properties, thus providing the possibility of various applications [3,4,5,6,7,8,9,10,11,12,13].

Technical thermoplastic composites are widely used in many industries, such as automotive, electrical engineering, robotics and production automation, and aerospace industries [14,15,16].

This wide use is closely related to the many advantages of these materials, such as low density, high stiffness and strength, good insulation properties, and corrosion resistance. In addition, these composites can be recycled, and the low weight allows for reduced CO_2_ emissions for transportation, which is very important for the environment and helps achieve sustainable development goals [17].

Technical thermoplastic composites are also increasingly being used in electrification products such as air-insulated medium-voltage switchgear components and poles used in medium-voltage circuit breakers and low-voltage disconnectors. They replace traditionally used hot chemically cured materials such as epoxy resins, which contain hazardous substances and have limited recycling possibilities [18].

The injection molding process, which is one of the main ways of processing technical thermoplastic composites, makes it possible to reduce the production cycle time greatly (from tens of minutes to tens of seconds) while achieving a high repeatability with the possibility of introducing partially or fully automated production [19].

Thermoplastic composites present high stiffness and strength due to using a filler, usually in the form of fibers. The introduction of fibers turns an isotropic thermoplastic material into an anisotropic composite with properties that vary depending on the direction of orientation of the fibers in it. Anisotropy refers to mechanical, thermal, and electrical properties and has a direct impact on the functional properties of the products, as well as on the manufacturing process itself, due to changes in the rheological properties of the material. Fiber orientation depends on the melt flow direction in the mold cavity during the injection molding process. The melt flow has a fountain-like character, and the accompanying shear effect at the boundary of the near-wall layer causes the fibers to be oriented in the flow direction. The fibers are oriented only in the vicinity of the near-wall layer, while the near-wall layer itself and the core of the part are characterized by a random arrangement of fibers [20].

A very interesting alternative to fiberglass infill is basalt fibers, which are produced from basalt rock and in a similar way to the fiberglass manufacturing process. Basalt fibers have a number of advantages over glass fibers, including higher tensile strength and greater resistance to weathering, alkalis, and acids [21].

Therefore, their application in technical thermoplastic composites can bring tangible benefits in improved mechanical properties [22] while increasing aging resistance, making them significant in products in electrification.

One of the challenges with implementing engineered thermoplastic composites in products for electrification is forming bonding lines when multiple injection points are used or when the two-particle stream is divided by an obstacle, forming a hole in the product. At the joining line, a significantly reduced strength is observed in the composite and is related to the discontinuity of the fiber structure and the formation of micro-carb on the surface of the product. Based on available studies, it can be expected that increasing the injection mold’s surface temperature through induction heating will reduce the negative impact of the joining line on the properties of thermoplastic composites [23,24].

Polyamide is a construction material most often used in various industries. This use is related to its properties. Polyamide is characterized by high strength properties while maintaining very good impact strength; it is an electrical insulator, a material resistant to elevated temperatures, and has excellent tribological properties. All of these characteristics promote this material to applications in the automotive, textile, engineering, construction, packaging, electronics, and electrical engineering industries [25].

Polyamides represent a group of polymeric materials that include, for example, the most widely used PA 6 and PA 46. In recent years, however, the most common polyamide is found in the form of fiber-reinforced composites. Fiberglass-reinforced polyamide is characterized by high stiffness and strength while maintaining good material deformability [26,27]. Another example of fibers used on a large scale is carbon fibers. Polyamide–carbon fiber composites are characterized by low weight, high strength properties, good fatigue, and corrosion resistance. They also show very good tribological properties, with low wear and friction coefficient [28].

Polyamide (PA) reinforced with short basalt fibers was also tested. The reinforcement contents were 5, 10, 15, 20, 30, and 40% by weight. The basalt fibers were characterized by a 3–4 mm length and a diameter of 13 μm. The addition of basalt fibers to polyamide improved tribological properties. The best friction and wear coefficient properties were obtained for the 10% basalt fiber compositions. It was found that the values of the friction coefficient and wear rate depended on the test conditions [29]. The presented examples of research on composites with basalt fibers show interest in this type of reinforcement in thermoplastic materials, but it should be mentioned that these fibers in continuous form are mainly implemented in duroplastic materials such as epoxy, polyester, vinylester, or phenyl resins [30,31,32,33,34,35,36].

There is growing interest in bio-based polymers being a viable alternative to their petrochemical counterparts [22,37].

All major plastics manufacturers, e.g., BASF, EMS-GRIVORY, DSM, DuPont, and Arkema, have bio-based polymers in their portfolio. Biopolymers such as PA and PET have real potential for use as a base for the thermoplastic engineering of composites in products for electrification. Biopolyamide of biological origin is most often produced from castor oil. It is widely used due to its excellent strength properties and chemical and thermal resistance. In addition, biopolyamides are characterized by lower water absorption than petrochemical polyamides [38].

Scientific research on biopolyamide-based composites indicates that synthetic, mineral, and natural fibers can be successfully used as reinforcing fibers. Examples include carbon, glass, and cellulose fibers. Each type of reinforcement provides slightly different properties, so composites of this type offer a wide range of properties and applications. For example, carbon fibers, in addition to the obvious reinforcement of the material, also provide better tribological properties by reducing the wear rate of the composite material. In contrast, glass fibers enhance the ability to absorb energy during dynamic impact, reducing the brittleness of the material [39,40,41,42].

Increasingly, polymeric materials are being used in the energy industry. In the work by Karki et al., an overview of composites and nanocomposites based on polymers that can be used in the power industry is presented. The advantages of polymeric materials are their unique properties like high resistance to degradation and, if required, biodegradability, ease of processing, low density, and low cost of production, all of which predispose them to energy generation and storage systems. As for potential applications, hybrid electric cars, high-power motors, radars, or materials used in the production of wind or water energy, as well as microelectronic systems using polymer capacitors, have been indicated. The most used polymeric materials are polyethylene naphthalate (PEN), polyphenylene sulfide (PPS), polyethylene terephthalate (PET) and polyimide (PI). In contrast, nylon 6, polyvinylidene fluoride (PVDF), cross-linked polyethylene (XLPE), and epoxy resins, both unfilled and filled with various fillers, are used as composite matrices. Examples of applications include portable electronics, adaptable pressure sensors, energy storage systems, water purification, and gas separation [43].

The undertaken research aims to evaluate the possibility of replacing petroleum-based polyamide 6.6 with bio-based polyamide 4.10, providing a 30% lower CO_2_ emission, i.e., reducing the environmental impact. The research considers basalt fibers, which have not been characterized in combination with polyamide 4.10, and provides a comparison against glass fibers. 

## 2. Materials and Methods

Keeping in mind the technical requirements for insulating materials, bio-based polyamide 4.10 was selected as the matrix material for the preparation of glass and basalt fiber composites. It is partially made (68%) from renewable sources (castor oil). The properties of applied PA4.10 were as follows: density, 1.16 g/cm^3^ and melting temperature, (10°/min) 250 °C.

The selected reference materials were as follows: (1) 60% bio-based polyamide 4.10 with 30% glass fiber content with density = 1.4 g/cm^3^ and melting temperature (10°/min) = 250 °C; and (2) polyamide 6.6 with 50% glass fiber content with density = 1.56 g/cm^3^ and melting temperature (10°/min) = 260 °C. 

The reinforcing additives were the following fibers suitable for polyamide thermoplastics:

(1) Glass fibers with a diameter of 10 μm and nominal chop length of 4.5 mm—Nippon Electric Glass ChopVantage HP 3610 (Otsu, Japan), with the following basic properties: density equal to 2.54 g/cm^3^, tensile strength approximately equal to 2.4 GPa, and Young’s modulus approximately equal to 76 GPa;
(2) Basalt fibers with a diameter of 13 μm and nominal chop length of 3.2 mm—BASALTEX (Kamenny Vek, Moscow, Russia) BSC13-3.2-KV02M with the following basic properties: density equal to 2.67 g/cm^3^, tensile strength approximately equal to 2.7 GPa, and Young’s modulus approximately equal to 85 GPa.

The granules of the tested composites were produced based on bio-based polyamide 4.10 and glass or basalt fibers in proportions of 15%, 30%, and 50%. Table 1 presents list of manufactured materials. The compounding process was realized on a laboratory twin-screw extruder Steer Omega 20H. Extrusion parameters were selected based on the recommendations of the plastic manufacturer and presented in Table 2. A tendency to aggregate basalt fibers in the feeder was observed during dosing of basalt fibers, which may affect dosing efficiency.

Samples for testing mechanical properties according to ISO 527 [44] were produced on a KraussMaffei injection molding machine at the Faculty of Materials Engineering and Physics, Krakow University of Technology. The process parameters were selected according to the material manufacturer’s recommendation and are summarized in Table 3.

One of the required characteristics of polymer composite materials is their low density, concerning their strength properties. In the present study, density was determined by a hydrostatic method using a RADWAG WAS 22W balance (Radom, Poland). Basic processing properties were determined using the melt flow rate. The test was carried out on a Zwick-Roell Mflow apparatus. The parameters included a temperature of 275 °C and a load of 5 kg. The testing and evaluation of the performance of plastics and polymer-based composites seem to be routine in an era of widespread use of these materials and, thus, not difficult. Still, basic strength tests indicate the direction of further research on material modification. Hence, the authors’ first steps were to determine the strength properties under static and dynamic conditions. Mechanical properties were tested by a static tensile test (PN-EN ISO 527-1:2019 [44]) on an MTS Criterion Model 43 universal testing machine (MTS System Corp., Eden Prairie, MN, USA) with a measuring range of up to 30 kN using an MTS axial extensometer. The test speed was set at 2 mm/min. The first loops of mechanical hysteresis were determined to visualize the viscoelastic nature of the fabricated composites. The tests were carried out on a Shimadzu AGS-X strength machine with software that allowed energy dispersion analysis (Autograph Trapezium XThe loading and unloading speed were 100 mm/min. 

## 3. Results

### 3.1. Physical and Mechanical Properties

**Figure 1:**
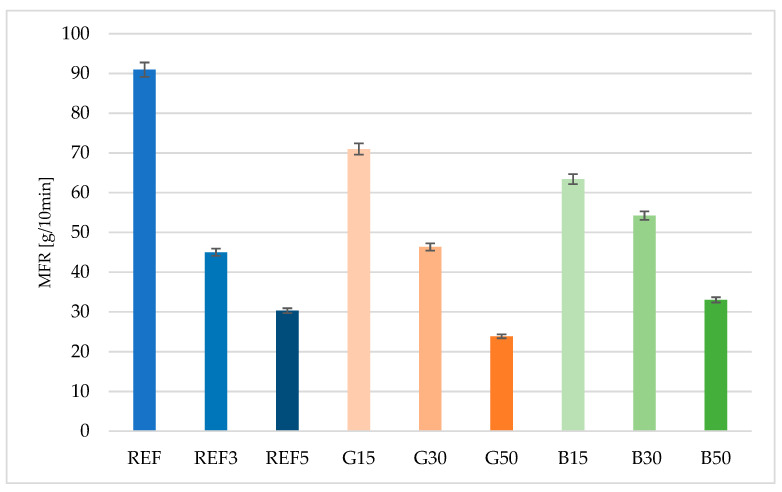
Comparison of the values of the mass flow rate.

Based on bio-based polyamide 4.10 and glass and basalt fibers, pellets of thermoplastic composites with 15%, 30%, and 50% fiber concentration were prepared. Figure 1 shows the results of melt flow coefficient measurements (275 °C and 5 kg) for the tested materials, considering the reference composite, i.e., commercial product. As the fiber concentration increased, the melt flow coefficient decreased, with lower melt flow coefficient values obtained for concentrations of 30% and 50% for the glass fiber. Similar melt flow coefficient values were obtained for the composite with 30% glass fiber doped under laboratory conditions (46.3 g/10 min; SDV 0.5) and the material supplied by the manufacturer (45.4 g/10 min; SDV 1.4), indicating a correct granular extrusion process.

For selected compositions, fiber weight percentage and fiber length tests were performed. The results are shown in Table 4. Analysis of the length distribution of glass fibers after the extrusion process indicated greater fiber fragmentation in the pellets obtained in the process involving the laboratory extruder than in the pellets supplied by the manufacturer, both for 30% and 50% concentrations. Basalt fibers underwent less fragmentation during the blending process compared to glass fibers.

From the prepared thermoplastic composite granules with 15%, 30%, and 50% glass or basalt fiber content, strength samples were produced in the form of paddles and beams for mechanical testing. Dry samples and those conditioned in a climate chamber were tested. The basic strength properties are shown in Table 5.

Reinforcement of materials is associated with the application of a second component to the matrix material. Properly carried out reinforcement processes increase the mechanical strength and stiffness of materials relative to the base material, but this does not mean that all properties are improved, as others, such as tribological or thermal properties, may remain unchanged, improve, or deteriorate. The efficiency of the applied reinforcement largely depends on the geometry of the fibers, i.e., the critical length (length-to-diameter ratio) and the interactions between the composite components. The highest strength properties are obtained with unidirectionally oriented fibers with a strongly developed reinforcement surface. In the case of injection molding of composites, it is difficult to achieve unidirectional fiber alignment due to fountain flow, and the fiber orientation inside is quite random [46].

**Figure 2:**
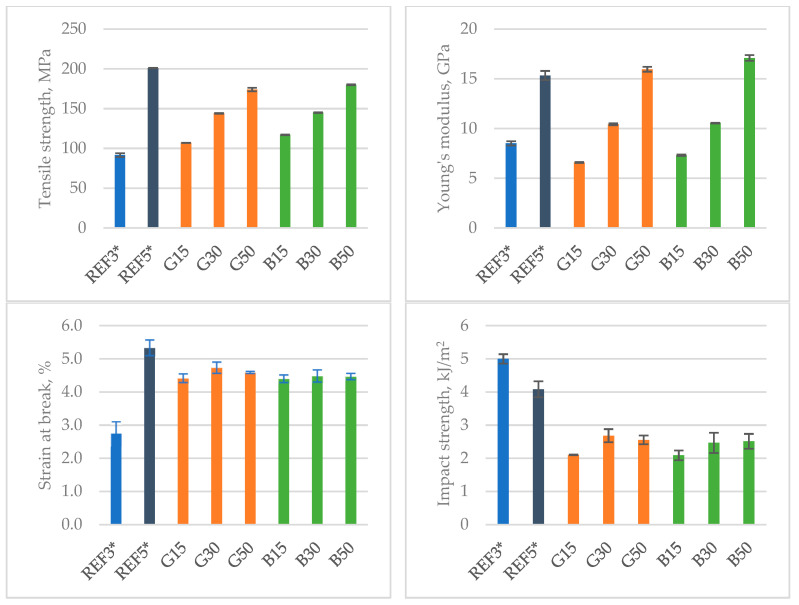
Mechanical properties of tested materials.

Figure 2 compares the basic strength and plastic properties of the tested composites. Tensile values increased with increasing fiber content in the composite matrix. Tensile strength values were similar regardless of whether glass or basalt fibers were used. However, it was observed that basalt fibers provided the highest stiffness to the composite relative to glass fibers in both biopolyamide and polyamide 6.6 matrix.

Comparable properties of composites with glass and basalt fibers may be related to the chemical similarity of both types of fibers. In the paper “Study on Mechanical Properties of Basalt Fibers Superior to E-glass Fibers”, five types of glass fiber types and five types of basalt fibers were compared. The test results showed that basalt fibers were characterized by higher mechanical properties than E-glass fibers. The strength of the fibers depended on the homogeneity of the fibers as well as the condition of the surface and internal defects. Studies showed that glass fibers were characterized by greater heterogeneity than basalt fibers, which resulted in their lower load-carrying capacity. In the presented work, all tested basalt fibers showed higher strength and modulus of elasticity in relation to E-glass fibers [46].

The strain at break value was maintained at a similar level of 4–4.5% and was slightly lower relative to commercially available polyamide 6.6 matrix materials. In the case of dynamic impact resistance, basalt fiber-modified materials showed an increase in impact strength with increasing fiber content, although the changes were small. Using more glass fibers causes an initial increase in energy absorption capacity, followed by a decrease at a fiber content of 50% by weight.

Similar test results were observed for other polymer composites, comparing the effectiveness of glass and basalt fibers. The tests showed that composites with basalt fibers showed higher flexural and compressive strength and provided a higher tensile modulus. In addition, fatigue strength and damping efficiency are also increased [47,48,49,50,51].

In addition, studies on biopolyamide composites reinforced with basalt fibers presented that after introducing basalt fibers into the polyamide matrix, the nucleation capacity increases, and the basalt fibers accelerate crystallization and eliminate the phenomenon of cold crystallization [52].

The function of the polymer matrix is to protect the reinforcement and transfer the load to it; however, due to the high stiffness of the fibers, the elongation at break value is much lower than that of the unreinforced material. From the mechanics of composites, it is known that at the fiber–matrix interface in the direction of the fiber ends, slippage increases; this is related to the increasing shear stress, which gradually decreases with increasing distance from the fiber surface. However, it is necessary to consider not only the fiber termination points but also the entire stresses along the fibers. In the case of plastic materials, there are significant differences in the distribution of shear stresses at the fiber–matrix interface, which greatly impact the strength and plastic properties of polymer composites [53].

Adhesion forces between components are also largely responsible for the reinforcement effect in polymer composites. Adhesion is caused by forces occurring at the reinforcement–matrix interface. Adhesion forces are physical forces resulting from the chemical buildup of the polymer matrix and the filler forming the composite. They can also be chemical actions that act by similarity of functional groups. Adhesion additionally depends on the degree of wetting, the magnitude of frictional forces at the interface, the action of shrinkage stress direction, the compactness and geometry of the filler, as well as defects in the form of voids and air bubbles. From the presented results, it can be concluded that the best bonding between components was characterized by the composite modified with particles of the smallest size, which is most likely related to the greater number of contact points at the particle–matrix interface. On the other hand, the introduction of larger particles with a dendritic structure caused a decrease in strength and plastic properties, which may be related to the formation of agglomerations and defects in the form of voids, which resulted in the weakening of the composite material [54].

In the case of the amount of reinforcement, it was found from experimental studies that there is a certain limiting fiber content beyond which the composite can be reinforced. Below this value, the fibers embedded in the polymer matrix cause stress concentrations, acting as internal notches that weaken the material. The introduction of more reinforcement causes stresses to be transferred to the fibers, and only after the fibers are broken are the stresses transferred to the matrix. As the proportion of fibers increases, both strength and Young’s modulus increase in the direction of the fiber axis, while the elongation at break value decreases [53].

**Figure 3:**
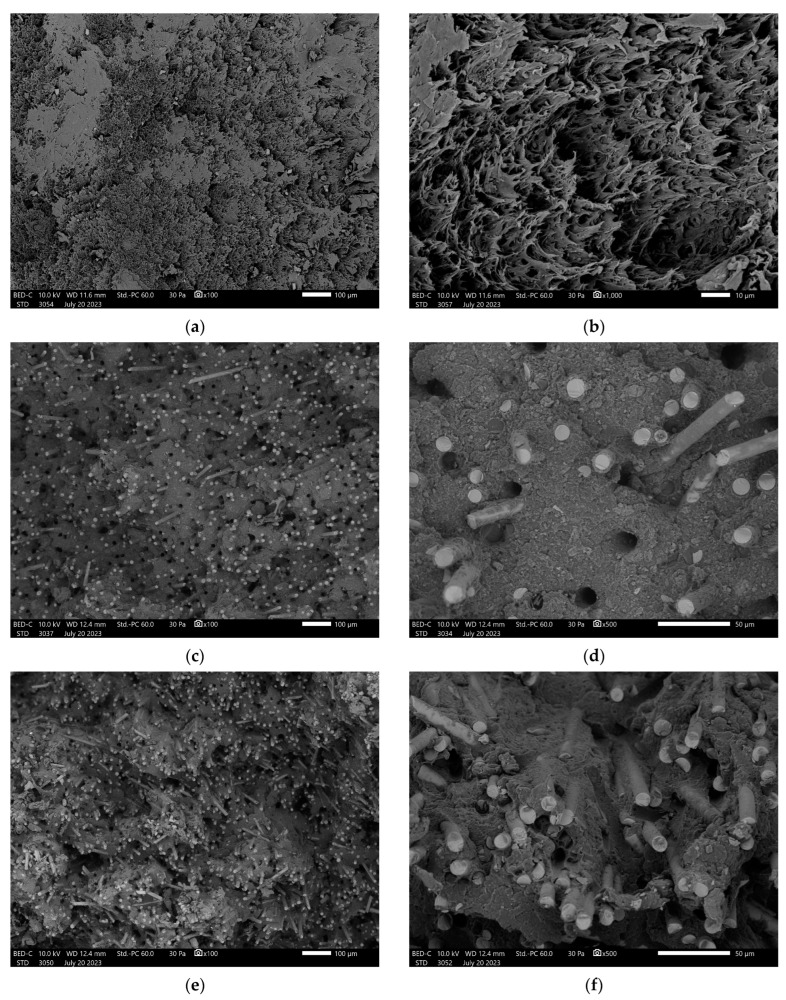

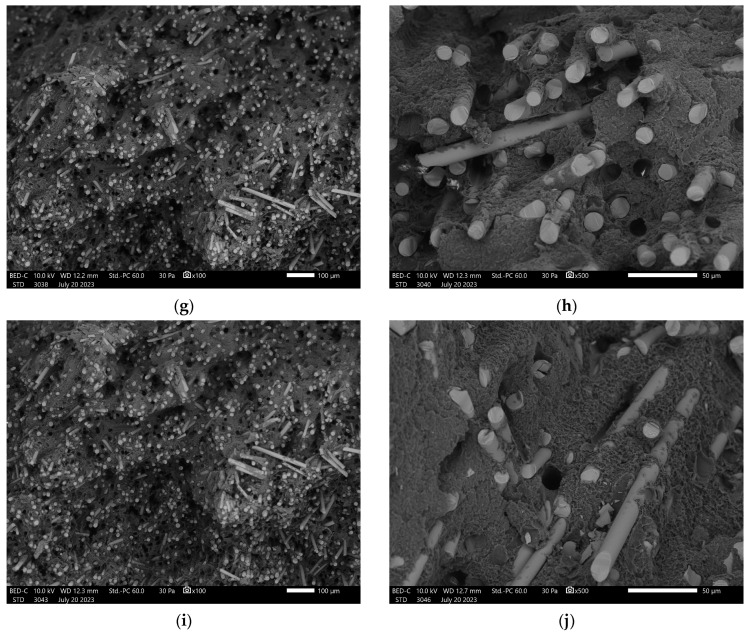
(**a**) REF × 100 magnification. (**b**) REF × 1000 magnification. (**c**) REF3 × 100 magnification. (**d**) REF3 × 500 magnification. (**e**) REF5. (**f**) REF5. (**g**) B50 × 100 magnification. (**h**) B50 × 500 magnification. (**i**) G50 × 100 magnification. (**j**) G50 × 500 magnification.

A comparison of microstructures is shown in Figure 3. The polymer matrix used to produce composites was characterized by a plastic fracture; the introduction of fibers changed the nature of cracking from plastic to brittle. The commercially used composites were characterized by an even distribution of fibers in the matrix, and the matrix wrapped around the fibers, which suggests good adhesion between the components. This suggests the use of an additional coupling agent that affects the connection between the phases. For the material containing 30% glass fibers (REF3), presented in Figure 3c,d, there were also significantly more places after the fibers were pulled compared to other materials. It can be concluded that both the pull-out mechanism and the mechanism of fiber breakage are dominant. This is confirmed by the results of strength tests because this material showed the lowest tensile strength and the highest impact strength. Composites with 50% glass fiber content (REF5) shown in Figure 3e,f were also characterized by very good adhesion; however, increasing the fiber density increases the share of the fiber cracking mechanism; the transfer of stress to the fibers is satisfactory. Accordingly, this material was characterized by the highest mechanical properties and very good impact strength.

The microstructures of the composites produced for the purposes of this research with both basalt (Figure 3g,h) and glass fibers (Figure 3i,j) were of comparable character; both cracking by the fibers and pulling the fibers out of the matrix were mechanisms of decohesion. Both glass and basalt fibers showed moderate adhesion to the polymer matrix. These similarities are related to the similar chemical composition of basalt and glass fibers. Observations also confirm the results obtained from strength tests, where these properties were at a comparable level.

**Figure 4:**
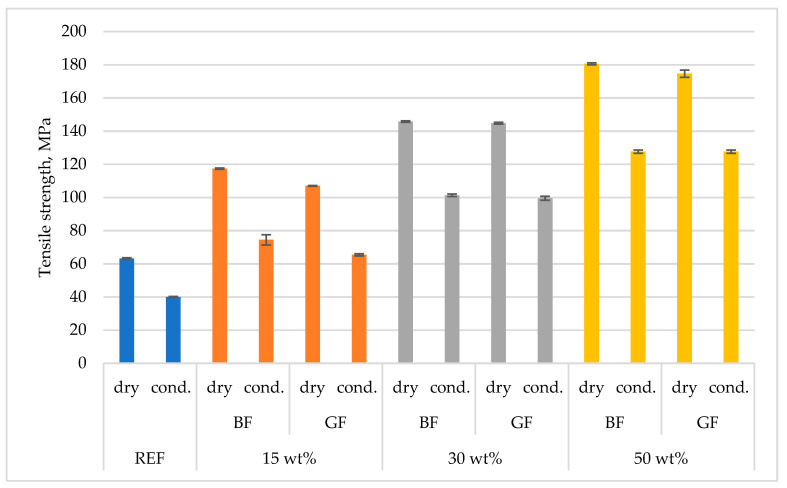
Comparison of tensile strength between different test conditions and different types of reinforcement.

**Figure 5:**
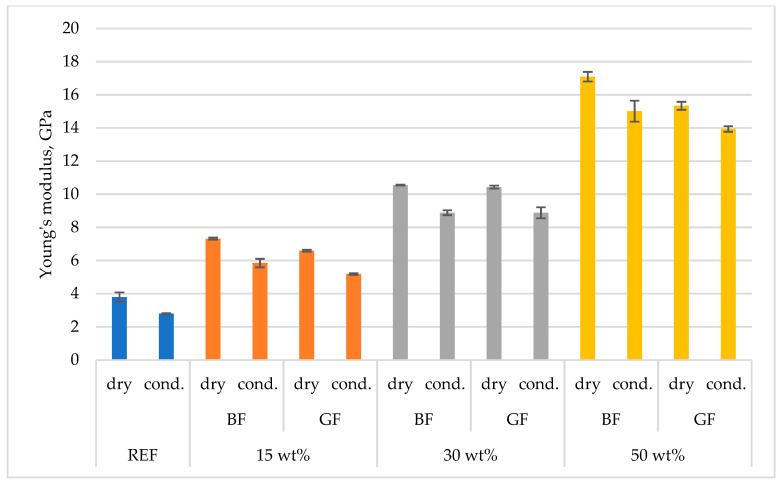
Comparison of tensile modulus between different test conditions and different types of reinforcement.

Strength tests were conducted on the materials in the dry and conditioned states. Figure 4 and Figure 5 show the differences resulting from the different states of the samples. Basalt fiber-based composites showed a slightly higher tensile strength. A lesser effect of moisture (conditioning) on the reduction in the elastic modulus was also observed for PA4.10 than for PA6.6, which is very important for products in electrification and is in accordance with the material manufacturer’s declarations [55]. In the case of composites containing mineral reinforcing fillers, the effect of the water environment took on a slightly different nature. The hydrophobic nature of mineral fibers has been confirmed in scientific studies. These fibers are characterized by low water absorption. The polymer’s propensity to absorb moisture decreases as the fiber content increases due to the reduction of the hygroscopic phase in the matrix as the fiber content increases. However, the type of fiber can change the water absorption after the occurrence of capillarity and affect the water content and absorption rate. Moisture penetration in a polymer composite along fibers is 100–400 faster than through the matrix; absorption of external moisture causes some plasticization of the material. Water particles can reduce the internal friction between the components of the composite and penetrate the matrix material, affecting the polymer chains [56].

### 3.2. Modeling of Effective Properties

The obtained empirical elastic modulus values were compared with calculated modulus values for different filler shares based on the rule of mixture (ROM) model and using the semi-empirical Halpin–Tsai approach [57]. An important element in the design of a material is the theoretical determination of its basic strength properties such as elastic modulus or tensile strength, which is made possible by various types of mathematical models, but it should also be considered that properties on a macro scale are the result of the properties of each component and the combination between the components, loading conditions, and environmental conditions [53].

One frequently used model for predicting strength properties is the Halpin–Tsai model [58]. This model, when using short fibers with random orientation, is as follows:

(1) $$E_{c}=E_{m}\left[\frac{3}{8}\left(\frac{1+\zeta \eta_{L}\phi_{f}}{1-\eta_{L}\phi_{f}}\right)+\frac{5}{8}\left(\frac{1+2\eta_{T}\phi_{f}}{1-\eta_{T}\phi_{f}}\right)\right]$$

where directional parameters $\eta_{L}$ and $\eta_{T}$ are:

(2) $$\eta_{L}=\frac{E_{f}/E_{m}-1}{E_{f}/E_{m}+\zeta }$$

(3) $$\eta_{T}=\frac{E_{f}/E_{m}-1}{E_{f}/E_{m}+2}$$

(4) ζ=2l/d

The parameter ζ describes the geometrical relationship between the fibers’ length and diameter. 

Another very simple model is the rule of mixture (ROM) model [51]:

$$E_{C}=V_{f}E_{f}+\left(1-V_{f}\right)E_{m}$$

where: 

$E_{C}$—Young’s modulus of composite;

$E_{f}$—Young’s modulus of fiber;

$E_{m}$—Young’s modulus of matrix;

$V_{f}$—Fiber volume fraction.

**Figure 6:**
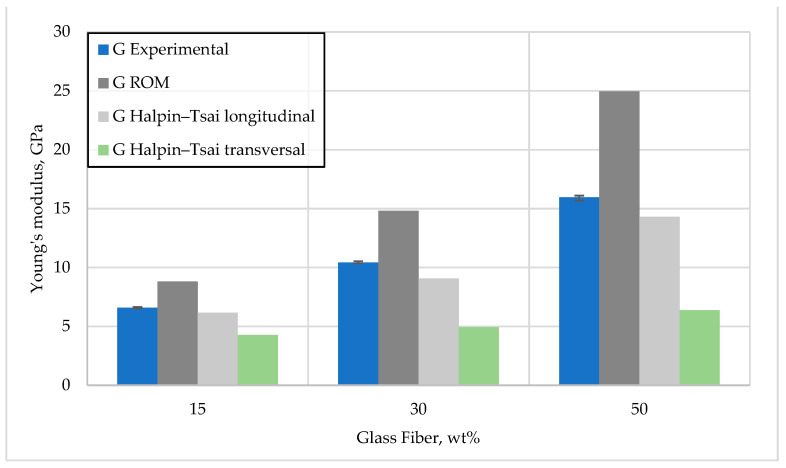
Comparison of Young’s modulus for glass fiber reinforcement—experimental and model results.

**Figure 7:**
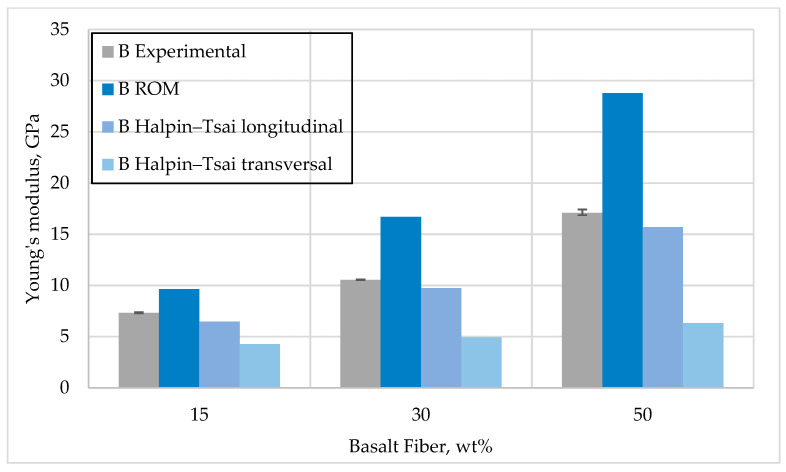
Comparison of Young’s modulus for basalt fiber reinforcement—experimental and model results.

Figure 6 and Figure 7 show basalt and glass fiber-reinforced composites’ measured and calculated elastic modulus values. The semi-empirical Halpin–Tsai model provides the best estimate of the post-longitudinal elastic modulus.

The results from comparing the obtained experimental values with the model values qualitatively coincide with the H–T model but differ slightly in quantity, which may be due to the adhesion procedures between the composite components. In the case of the rule of mixture model, these values are quite inflated, which is due to the rather simple parameters that this model considers.

### 3.3. Energy Dissipation

Determination of the initial mechanical hysteresis loops of low-cycle load tests makes it possible to determine the dissipation energy occurring in the material during cyclic load changes. Unmodified materials dissipate energy due to internal friction between polymer chains, generating thermal energy. The method can also be used to analyze interactions between components in composite materials, as the energy delivered to the system is also dissipated due to the relaxation of internal stresses generated during the manufacturing process, as well as to eliminate local stresses related to the connection of components such as pull-out effects of fibers and at other locations where defects occur in the form of notches and discontinuities [59].

**Figure 8:**
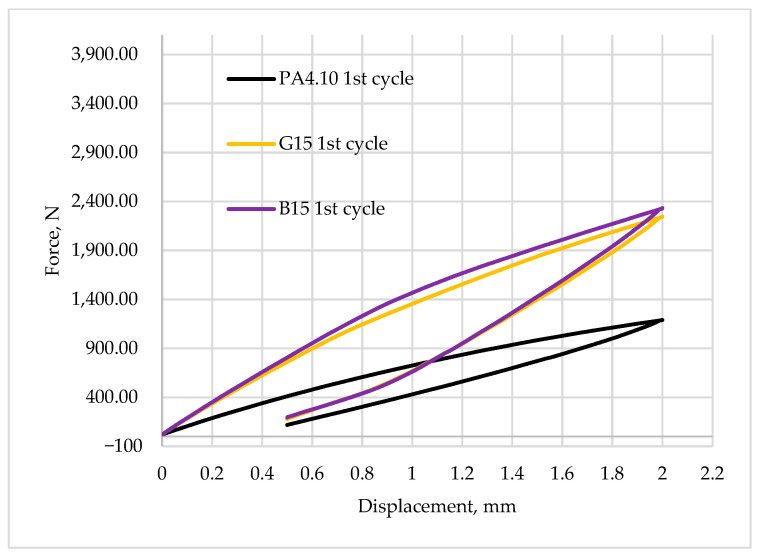
The first hysteresis loop for composites reinforced by 10 wt.% of fibers and for base material.

**Figure 9:**
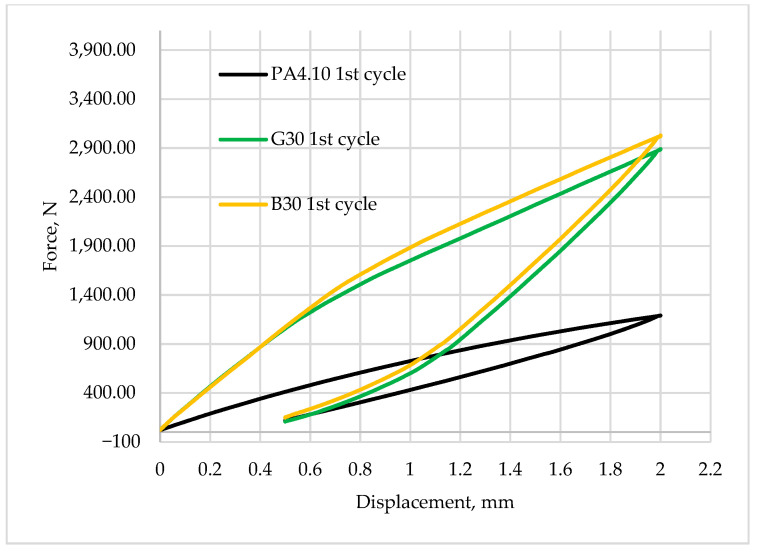
The first hysteresis loop for composites reinforced by 30 wt.% of fibers and for base material.

**Figure 10:**
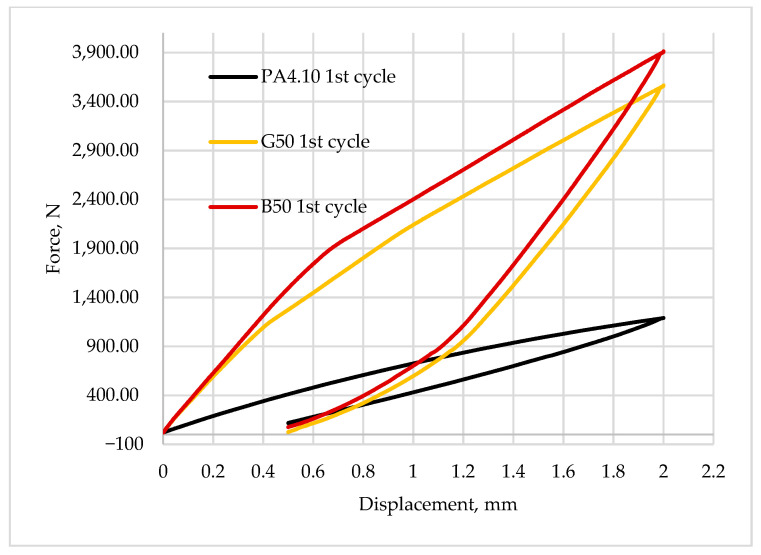
The first hysteresis loop for composites reinforced by 50 wt.% of fibers and for base material.

**Figure 11:**
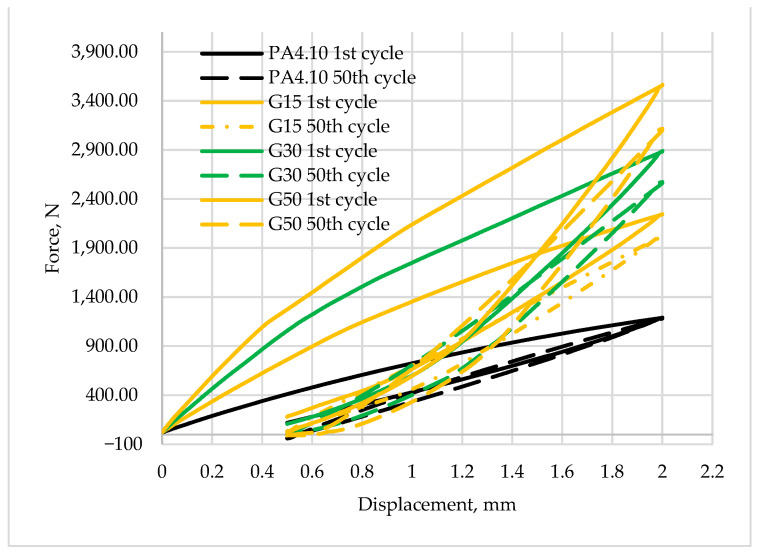
The first and last hysteresis loops for composites reinforced by glass fibers and for base material.

**Figure 12:**
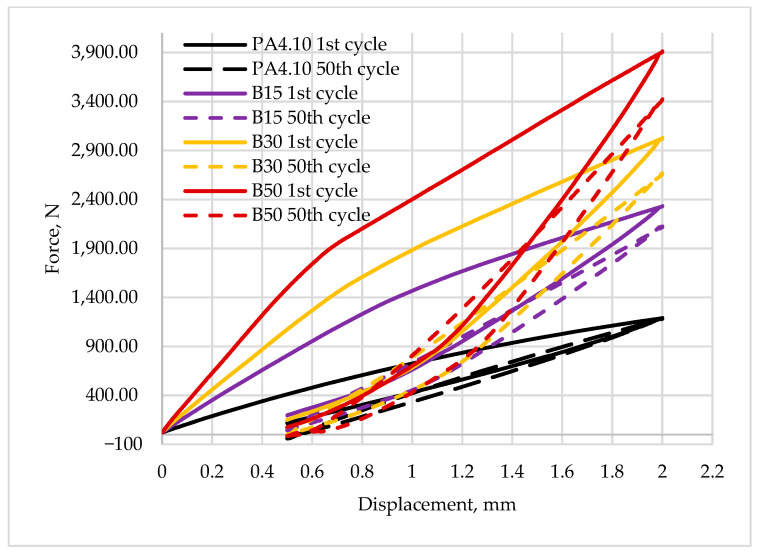
The first and last hysteresis loops for composites reinforced by basalt fibers and for base material.

Figure 8, Figure 9, Figure 10, Figure 11 and Figure 12 show the recorded hysteresis loops. Figure 8 shows the first hysteresis loops for composites containing a 15% fiber content and a base material.

Figure 9 and Figure 10 show a comparison of the recorded first hysteresis loops for composites reinforced with 30 and 50% fiber contents. The presented results indicate higher values of the force required to deform the material by a given value for composites with basalt fibers, which present a greater ability to strengthen the material. Figure 11 and Figure 12 show a comparison of the first and last hysteresis loops. It was observed that the force required to deform the material decreases with an increasing number of cycles, suggesting a cyclic weakening of the materials.

Similar test results were observed for composites based on polyacetal and polypropylene modified with glass fibers. Previous authors have pointed to three possible relationships occurring in materials during cyclic deformation. When the peak loop tension increases with an increasing number of cycles, the width of the hysteresis loop decreases, and the material is cyclically strengthened. If peak stresses decrease with increasing numbers of cycles until a steady state occurs, cyclical weakening of the material appears, as observed in the presented test results. A third possibility is that there are no significant changes in peak stresses depending on cyclic loads, in which case the material is considered cyclically stable [60,61].

**Figure 13:**
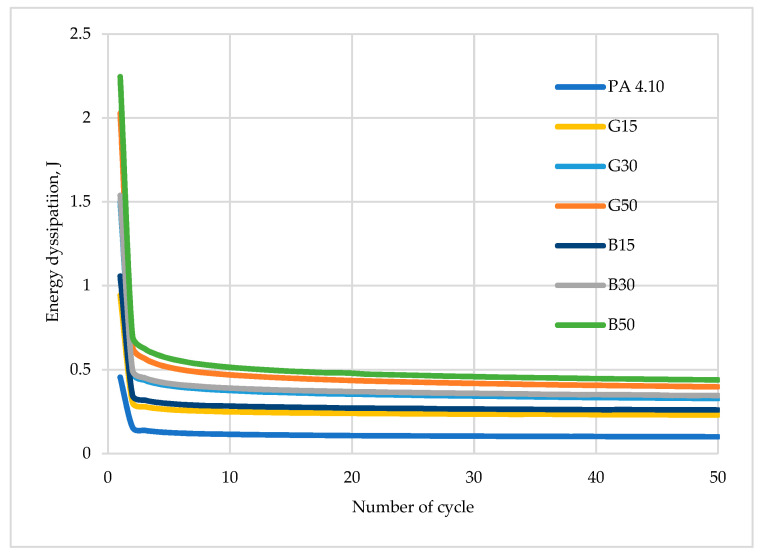
Energy dissipation in relation to number of cycles.

It can be noted that the first hysteresis loops for the composites are decidedly larger, and the dissipated energy (Table 6) is in the range of 0.9–1.0 J and about 0.45 J for the matrix material. Similar conclusions can be drawn for other materials with 30 and 50% fiber contents. It is also noted that the value of propagation energy is slightly higher for materials modified with basalt fibers. As the number of interacting cycles increases, the dissipated energy stabilizes after only about four or five cycles and at the fiftieth cycle, the load–unload energy varies in the range of 0.2–0.3 J, with a continuing trend of higher values for composites with basalt fibers. The increased areas of the hysteresis loops may suggest that the energy supplied to the system was dissipated into the interactions between the composite components. Similar results have been observed for other composite materials modified with both natural and synthetic fibers and particles [22,62,63]. A theory was developed by Cieszynski and Topolinski, who presented a relationship between the energy of dispersion and the properties of multiphase materials in their study. The theory is based on the finding that in the first cycles of loading, locally occurring stresses in interfacial areas undergo a relaxation process, and the maximally stressed areas successively crack, which is registered in the form of hysteresis loops due to the use of supplied energy for relaxation and cracking processes [64]. The registration of dozens of hysteresis loops and determination of the dissipation energy confirm the presented theory as after the first few cycles, the dissipation decreases and eventually stabilizes at a relatively low level, as shown in Figure 13.

## 4. Conclusions

This study aimed to produce and investigate the basic mechanical and low-cycle dynamic properties of biopolymer matrix composites. The use of glass and basalt fibers in concentrations of 15, 30, and 50% by weight was compared. The obtained test results were compared with commercially available composites based on polyamide 6.6 matrix with 50% glass fiber content and biopolyamide 4.10 reinforced with 30% glass fiber content. The results of the study showed that the use of biopolyamide 4.10 as a matrix of structural materials is satisfactory, demonstrating ease of modification and obtaining very high strength properties, higher than those of commercially available composites, allowing us to conclude that bio-based materials can successfully replace materials of petroleum origin. The presented analysis of the obtained test results additionally indicates that basalt fibers comprise a reinforcement that provides very high strength properties, higher than that of used glass fibers at the same level of composite filling. In the presented studies, the basalt fibers were characterized by a shorter length compared to glass fibers, which only confirms their high efficiency as reinforcements and a fairly good connection with the matrix in the form of biopolyamide. The analysis of the structure showed that both glass fibers and basalt fibers present moderate adhesion to the polyamide matrix; however, it should be noted that no coupling additive was introduced, and the obtained results are satisfactory. Additional introduction of adhesion promoters would certainly allow for even better strength results, which indicates the great potential of this type of composite in advanced applications.

## Figures and Tables

**Table 1:** Description of produced composites.

Description	Index	Matrix	Reinforcement	% wt. Share
PA410 + 15% Glass fiber	G15	PA410	ChopVantage HP3610	15
PA410 + 30% Glass fiber	G30			30
PA410 + 50% Glass fiber	G50			50
PA410 + 15% Basalt fiber	B15		BSC13-3.2-KV02M	15
PA410 + 30% Basalt fiber	B30			30
PA410 + 50% Basalt fiber	B50			50

**Table 2:** Parameters of extrusion process.

Index	RPM	Torque, %	Feeder, TS	RPM Side	Feeder GF/BF	Temperature of Cylinder, °C	RPM Reception	RPM Cutting
G15	90	55	20	100	5	260/250	40	30
G30		82	25	100	15	260/250	45	30
G50		82	20	100	25	260/250	40	30
B15		69	25	100	20/10	260/250	40	30
B30		59	20	100	35/10	260/250	35	30
B50		56	15	100-150-460	65/10	260/250	33	25

**Table 3:** Parameters of injection mounding.

I Zone, °C	II Zone, °C	III Zone, °C	IV ZONE, °C	Nozzle, °C	Pressure of Injection, Bar	Pressure of Packing, Bar	Mold, °C
260	265	265	275	290	1400	500	80

**Table 4:** Filler content according to ISO 1172 [45] and average glass fiber length.

Index	Filler Content, wt%	Arithmetic Mean Length, µm	Weighted Mean Length, µm
REF3*	36.3	375	429
REF5*	49.3	312	351
G15	14.7	226	264
G30	26.7	252	284
G50	48.0	220	261
B15	17.7	348	399
B30	30.6	389	453
B50	49.3	328	365

REF3* Commercially available bio-based polyamide 4.10 with 30% glass fiber content. REF5* Commercially available polyamide 6.6 with 50% glass fiber content.

**Table 5:** Basic mechanical properties of tested materials.

Index	Tensile Strength, MPa	Tensile Modulus, MPa	Strain at Break, %	Impact Strength, kJ/m^2^
REF	40.3 ± 0.3	2765 ± 45	5.53 ± 0.8	±
REF3*	91.6 ± 2.3	8502 ± 210	2.8 ± 0.4	5.000 ± 0.140
REF5*	201.1 ± 0.4	15,333 ± 458	5.3 ± 0.2	4.082 ± 0.240
G15	107.0 ± 0.2	6587 ± 57	4.4 ± 0.1	2.102 ± 0.012
G30	144.8 ± 0.5	10,432 ± 94	4.7 ± 0.2	2.683 ± 0.197
G50	174.6 ± 2.1	15,955 ± 241	4.6 ± 0.1	2.555 ± 0.133
B15	117.4 ± 0.4	7313 ± 65	4.4 ± 0.1	2.090 ± 0.146
B30	145.8 ± 0.5	10,552 ± 34	4.5 ± 0.2	2.466 ± 0.304
B50	180.5 ± 0.6	17,091 ± 291	4.5 ± 0.1	2.513 ± 0.223

REF3* Commercially available bio-based polyamide 4.10 with 30% glass fiber content; REF5* Commercially available polyamide 6.6 with 50% glass fiber content.

**Table 6:** Energy dissipation for tested materials.

Index	Energy Dissipation, J		
	1st Cycle	2nd Cycle	50th Cycle
REF	0.454	0.157	0.099
G15	0.943	0.306	0.229
G30	1.499	0.483	0.327
G50	2.026	0.639	0.397
B15	1.057	0.349	0.259
B30	1.537	0.498	0.346
B50	2.245	0.702	0.438

## Data Availability

Data available on request due to restrictions eg privacy or ethical. The data presented in this study are available on request from the corresponding author. The data are not publicly available due to trade secret of the company.
